# Unlocking Health Literacy: The Ultimate Guide to Hypertension Education From ChatGPT Versus Google Gemini

**DOI:** 10.7759/cureus.59898

**Published:** 2024-05-08

**Authors:** Thomas J Lee, Daniel J Campbell, Shriya Patel, Afif Hossain, Navid Radfar, Emaad Siddiqui, Julius M Gardin

**Affiliations:** 1 Department of Medicine, Rutgers University New Jersey Medical School, Newark, USA; 2 Department of Otolaryngology–Head and Neck Surgery, Thomas Jefferson University Hospital, Philadelphia, USA; 3 Department of Cardiology, Rutgers University New Jersey Medical School, Newark, USA

**Keywords:** google gemini, chatgpt, hypertension, patient education, artificial intelligence

## Abstract

Background

Google Gemini (Google, Mountain View, CA) represents the latest advances in the realm of artificial intelligence (AI) and has garnered attention due to its capabilities similar to the increasingly popular ChatGPT (OpenAI, San Francisco, CA). Accurate dissemination of information on common conditions such as hypertension is critical for patient comprehension and management. Despite the ubiquity of AI, comparisons between ChatGPT and Gemini remain unexplored.

Methods

ChatGPT and Gemini were asked 52 questions derived from the American College of Cardiology’s (ACC) frequently asked questions on hypertension, following a specified prompt. Prompts included: no prompting (Form 1), patient-friendly prompting (Form 2), physician-level prompting (Form 3), and prompting for statistics/references (Form 4). Responses were scored as incorrect, partially correct, or correct. Flesch-Kincaid (FK) grade level and word count were recorded.

Results

Across all forms, scoring frequencies were as follows: 23 (5.5%) incorrect, 162 (38.9%) partially correct, and 231 (55.5%) correct. ChatGPT showed higher rates of partially correct answers than Gemini (p = 0.0346). Physician-level prompts resulted in a higher word count across both platforms (p < 0.001). ChatGPT showed a higher FK grade level (p = 0.033) in physician-friendly prompting. Gemini exhibited a significantly higher mean word count (p < 0.001); however, ChatGPT had a higher FK grade level across all forms (p > 0.001).

Conclusion

To our knowledge, this study is the first to compare cardiology-related responses from ChatGPT and Gemini, two of the most popular AI chatbots. The grade level for most responses was collegiate level, which was above average for the National Institutes of Health (NIH) recommendations, but on par with most online medical information. Both chatbots responded with a high degree of accuracy, with inaccuracies being rare. Therefore, it is reasonable that cardiologists suggest either chatbot as a source of supplementary education.

## Introduction

Hypertension is the most common cardiovascular disease and the leading cause of cardiovascular mortality worldwide [1,2]. Over half of the world’s population has been diagnosed with hypertension, with a much greater percentage suspected, but not yet diagnosed with hypertension [3]. Furthermore, hypertension is a systemic disease contributing to adverse effects in multiple organ systems and overall lifestyle. Patient education plays an especially pivotal role in managing hypertension, given that disease treatment is inherently multi-factorial, involving medications, diet, exercise, or life stressors [4].

According to the National Cancer Institute’s (NCI) Health Information National Trends Survey (HINTS), 84.6% of the US adult population used the internet to look for health or medical information in 2022, with a number expected to rise in the coming decade [5]. The literature on the prevalence of utilizing artificial intelligence (AI) chatbots for medical education is poorly defined; however, the trend of utilization of AI chatbots has been steadily rising even after its initial exponential rise.

ChatGPT, an AI chatbot developed by OpenAI (San Francisco, CA) in November of 2022, has quickly gained widespread attention. From its inception, the site took five days to reach 1 million users and within two months, it had surpassed 100 million users [6]. In response to the rise of AI chatbots, Google (Mountain View, CA) released Gemini, a large language model similar to that of ChatGPT, on May 10, 2023. Within a few months, Gemini rose to become the primary competitor to ChatGPT [7,8].

Given AI’s burgeoning popularity and its potential for disseminating health information, evaluating the quality and accuracy of ChatGPT and Gemini is of paramount importance. We aimed to critically assess ChatGPT's responses to queries about one of the world’s most common diseases, hypertension. This study focuses on the accuracy, comprehensibility, and appropriateness of using AI responses for patient education. The purpose of this study is to guide healthcare professionals and patients in understanding the benefits and potential limitations of AI for patient education.

## Materials and methods

OpenAI’s ChatGPT and Google’s Gemini chatbots were prompted four times, then asked 52 questions from the 2017 American College of Cardiology’s (ACC) frequently asked questions on hypertension [9]. ChatGPT version 3.5 and Gemini version 1.0 (formerly known as Google Bard) were used for all responses. All questions were asked between the dates of September 6, 2023, and September 7, 2023.

Prompts were as follows: no prompt (Form 1), patient-friendly prompt (Form 2), physician-level prompt (Form 3), and prompting for statistics/references (Form 4). The prompts used are presented in Table 1. Responses were reviewed and scored as incorrect, partially correct, correct, or correct with references (perfect). Incorrect responses were designated if the response included any incorrect information or if responses included less than 50% of information from the ACC response answers. Partially correct answers included responses that had no incorrect information and included 50%-99% of the information from the ACC responses. Correct responses included all information from the ACC responses with any extra information being correct. Perfect responses included responses that met the criteria for correct responses and included references and/or statistics in the response. Proportions of responses at differing scores were compared using chi-square analysis. Tests were performed with an alpha set at 0.05.

**Table 1:** ChatGPT and Gemini prompts. Prompts provided to either ChatGPT or Gemini before asking questions.

Form number	Form name	ChatGPT/Gemini Prompt
1	No prompting	No prompting.
2	Patient-friendly prompting	I am a patient attempting to learn more about hypertension. I am going to ask you 52 questions pertaining to hypertension. Please use language that would be appropriate for my understanding, but do not compromise on the accuracy of your responses. Be as specific as possible in your answers.
3	Physician-level prompting	I am a board-certified physician attempting to learn the most up-to-date information on hypertension. I am going to ask you 52 questions pertaining to hypertension. Please use language that would be appropriate for my expert-level understanding of medical concepts. Be as specific as possible in your answers.
4	Prompting for statistics and references	I am going to ask you 52 questions pertaining to hypertension. For each answer you provide, make sure that you include statistics, numbers, or calculations that are relevant. Your answers should come from published medical literature, which you should cite within your answers.

For each response, the number of words, sentences, and syllables were collected to compute a Flesh-Kincaid (FK) grade level. This metric estimates the United States educational grade level required to understand the response, with higher grade levels indicating more complex language usage and is defined as:

$0.39( \frac{words}{sentences}) + 11.8 ( \frac{syllables}{words}) - 15.59$.

Values vary from 0 to 20, with the numerical value corresponding with the reading grade level (e.g., 12 would equal grade level 12). Significance between forms was calculated using a one-way ANOVA with an alpha of 0.05. Additionally, response length was recorded and significance was analyzed with a one-way ANOVA and an alpha set at 0.05. The significance for statistical analyses was set at p < 0.05. Statistics were run using Prism 10.0.2 (GraphPad Software, San Diego, CA).

## Results

**Figure 1:**
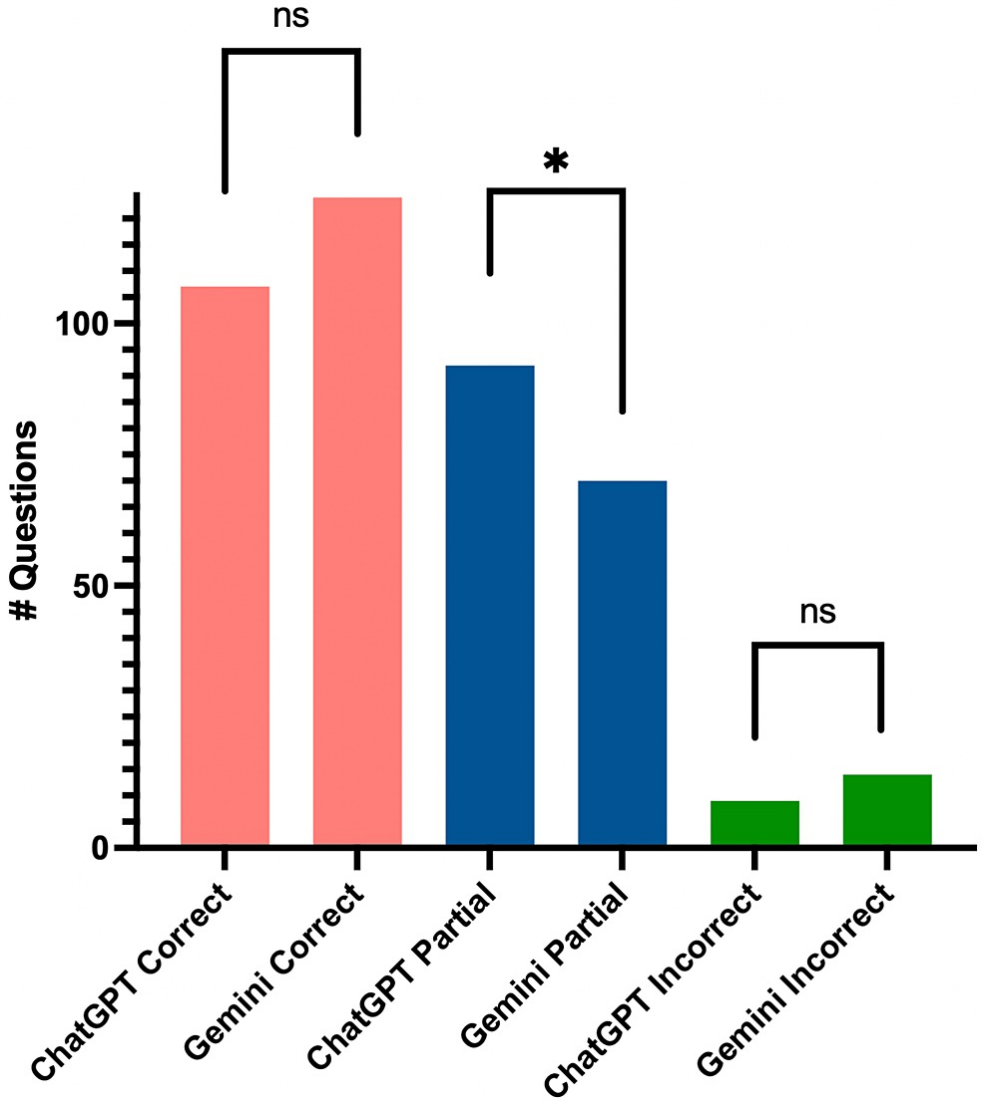
Correct, partially correct, and incorrect answers in ChatGPT and Gemini responses. Each bar shows the total number of correct, partially correct, or incorrect answers between all forms. Abbreviations: ns = no significance. * = p < 0.05.

Across all forms, scoring frequencies for ChatGPT were as follows: nine (4.33%) incorrect, 92 (44.23%) partially correct, and 107 (51.44%) correct. Scoring frequencies for Gemini were as follows: 14 (6.73%) incorrect, 70 (33.65%) partially correct, and 124 (59.62%) correct. Chi-squared analysis revealed the proportions of responses categorized as correct did not significantly differ between ChatGPT vs. Gemini (p = 0.11). However, ChatGPT was more likely to give a partially correct response when compared to Gemini (p = 0.035) (Figure 1).

**Figure 2:**
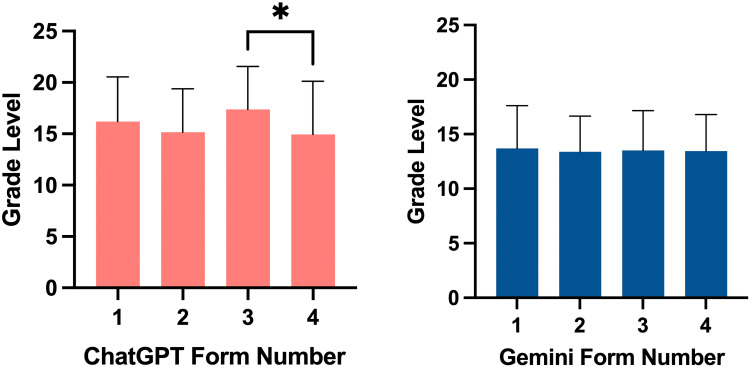
Grade reading level for ChatGPT and Gemini responses. * = p < 0.05.

FK scores for ChatGPT and Gemini can be found in Figure 2. ChatGPT’s mean FK grade reading level was as follows: Form 1 at 16.20 (±4.36), Form 2 at 15.15 (±4.25), Form 3 at 17.37 (±4.20), and Form 4 at 14.94 (±5.17). In ChatGPT responses, a significant difference was found between Form 3 and 4’s grade reading level (p = 0.033). Gemini’s mean FK grade reading level was as follows: Form 1 at 13.69 (±3.92), Form 2 at 13.38 (±3.27), Form 3 at 13.50 (±3.67), and Form 4 at 13.44 (±3.37). There was no significant difference in reading level between forms for Gemini’s responses. Overall ChatGPT’s responses had a higher grade reading level (15.92 ± 4.58) than Gemini’s responses (13.50 ± 3.54) (p < 0.0001).

**Figure 3:**
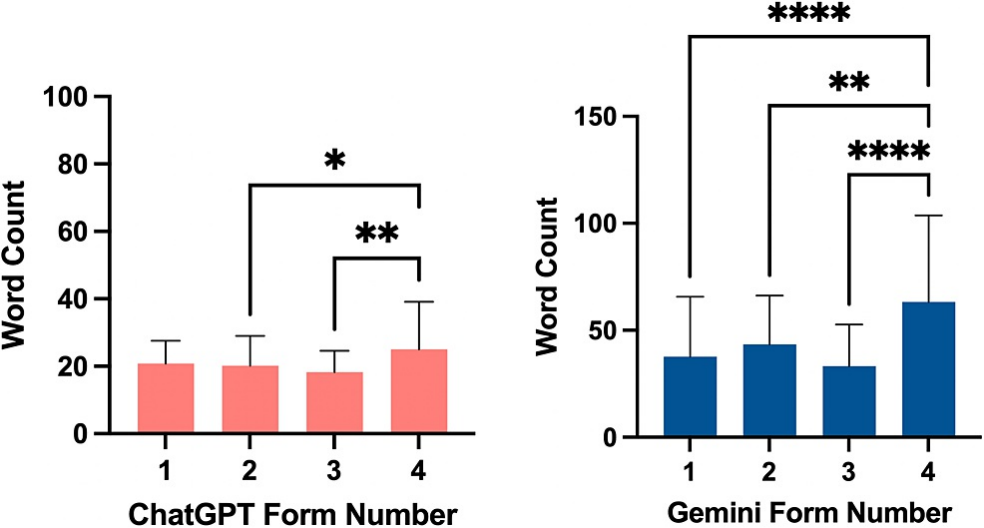
Word count for ChatGPT and Gemini responses. * = p < 0.05; ** = p < 0.01; **** = p < 0.0001.

Word count for ChatGPT and Gemini can be found in Figure 3. ChatGPT’s mean word count was as follows: Form 1 at 19.00 (±6.74), Form 2 at 19.00 (±8.84), Form 3 at 18.00 (±6.30), and Form 4 at 20.50 (±14.07). Gemini’s mean word count was as follows: Form 1 at 27.50 (±28.02), Form 2 at 38.00 (±22.81), Form 3 at 27.50 (±19.41), and Form 4 at 56.00 (±40.45). Overall Gemini’s responses had a higher word count (44.43 ± 30.82) than ChatGPT’s responses (21.08 ± 9.75) (p < 0.0001).

## Discussion

Since the inception of Google Gemini, comparisons to ChatGPT have been made, and there have been many speculations about which AI chatbot would be more accurate [10,11]. To date, few studies have objectively compared the accuracy of responses, with even fewer studies focusing on the medical field [12]. To our knowledge, this is the first study to compare the performance of two of the most popular AI chatbots, ChatGPT and Gemini, on cardiology-related topics.

Overall, both ChatGPT and Gemini provided accurate, but often partially complete, responses when responding to ACC’s frequently asked questions about hypertension. Even though only half the answers were deemed entirely "correct," this result was still seen positively. The AI chatbots' replies often contained more than 50% of the information, typically lacking just one element from the ACC's answers. The responses that would signify a large deficiency - incorrect or incomplete (greater than 50% missing) information - were only present in 5.5% of responses. This result was on par with many other studies that examined AI chatbot responses, generally ranging from 1% to 5% incorrect responses [13-16]. ChatGPT gave more partially correct answers than Gemini, while Gemini exhibited a non-significant trend to provide more correct responses than did ChatGPT. This could be in part because ChatGPT’s mean responses were 23 words shorter than Gemini’s responses, leaving less room for information.

These findings highlight the significance of effective prompting in optimizing the comprehension of responses from chatbots. We observed that variations in prompts led to differences in both the grade level and the word count of the responses. Similar to previous studies on artificial intelligence's handling of hypertension, effective prompting and active engagement are essential for achieving optimal outcomes [17].

ChatGPT had a higher mean grade reading level than Gemini, with an FK score of 15.92 versus 13.50, respectively. Although ChatGPT’s answers were less accurate, they were more succinct and used a higher grade reading level. The National Institutes of Health (NIH) recommends patient education material should be written at an 8th-grade reading level, which is lower than both ChatGPT’s approximate grade level (grade 15 - collegiate level), and Gemini’s approximate grade reading level (grade 13 - collegiate level) [18]. However, ChatGPT and Gemini’s grade levels are quite similar to many online sources of cardiology material. Academic websites pertaining to atrial fibrillation had a mean grade level of 13.05, while non-academic sites had a mean average of 11.64 [19]. This finding was mirrored in other medical specialties’ online reading material [20-23]. Therefore, while the two chatbots responded above the NIH-recommended grade level, the responses were on par with most online resources.

ChatGPT consistently had a lower average word count in its responses compared to Gemini, as noted earlier. Similar trends have been observed in other studies comparing the two chatbots' performance on health literacy, hinting that Gemini may naturally provide lengthier responses [24]. Notably, the word count for both chatbots remained fairly consistent across various query types with the exception of Form 4, which involves requesting statistics or research. This variation is likely due to the nature of the prompt, as requesting data and references typically necessitates the inclusion of more detailed information, such as citations, statistical figures, or mathematical equations.

While this study assesses responses objectively, it has its limitations, including the assumption of accurate patient inquiries. We did not assess the chatbots' reactions to false information. Also, patients have myriad ways to ask questions, potentially leading to responses not reviewed in this study. Future research should broaden the scope of inquiries and analyze the chatbots' handling of erroneous inputs.

## Conclusions

The analysis shows that AI chatbots like ChatGPT and Gemini can be valuable tools for augmenting patient education on topics such as hypertension. Both have demonstrated a strong ability to provide accurate answers. They might not include every nuance that the ACC offers, but they generally convey the necessary information with few errors. Therefore, it is sensible for medical professionals to suggest using ChatGPT or Gemini as educational resources if future studies continue to report positive responses. Nevertheless, one should recognize the minor possibility of encountering inaccuracies.
